# A Novel PDE10A Inhibitor for Tourette Syndrome and Other Movement Disorders

**DOI:** 10.3390/cells13141230

**Published:** 2024-07-22

**Authors:** Randall D. Marshall, Frank S. Menniti, Mark A. Tepper

**Affiliations:** 1 EuMentis Therapeutics Inc., 275 Grove Street, 2-400, Newton, MA 02466, USA; mtepper@eumentistx.com; 2 MindImmune Therapeutics, Inc., Kingston, RI 02881, USA; mennitifs@gmail.com; 3 The George & Anne Ryan Institute for Neuroscience, University of Rhode Island, Kingston, RI 02881, USA

**Keywords:** PDE10, pharmacology, toxicology, phase 1, proof of mechanism, PET

## Abstract

Background: Tourette syndrome is a neurodevelopmental movement disorder involving basal ganglia dysfunction. PDE10A inhibitors modulate signaling in the striatal basal ganglia nuclei and are thus of interest as potential therapeutics in treating Tourette syndrome and other movement disorders. Methods: The preclinical pharmacology and toxicology, human safety and tolerability, and human PET striatal enzyme occupancy data for the PDE10A inhibitor EM-221 are presented. Results: EM-221 inhibited PDE10A with an in vitro IC50 of 9 pM and was >100,000 selective vs. other PDEs and other CNS receptors and enzymes. In rats, at doses of 0.05–0.50 mg/kg, EM-221 reduced hyperlocomotion and the disruption of prepulse inhibition induced by MK-801, attenuated conditioned avoidance, and facilitated novel object recognition, consistent with PDE10A’s inhibition. EM-221 displayed no genotoxicity and was well tolerated up to 300 mg/kg in rats and 100 mg/kg in dogs. In single- and multiple-day ascending dose studies in healthy human volunteers, EM-221 was well tolerated up to 10 mg, with a maximum tolerated dose of 15 mg. PET imaging indicated that a PDE10A enzyme occupancy of up to 92.8% was achieved with a ~24 h half-life. Conclusions: The preclinical and clinical data presented here support the study of EM-221 in phase 2 trials of Tourette syndrome and other movement disorders.

## 1. Introduction

Tourette syndrome (TS) is a neurodevelopmental disorder that is characterized by the presence of tics, which are repetitive, stereotyped, and involuntary movements or vocalizations that persist for at least 1 year [1,2]. Tics tend to emerge before adolescence, with variable intensity and frequency, and can wane, disappear, or persist as the brain matures into young adulthood. A greater severity in childhood of TS symptoms and comorbid OCD and ADHD is a strong predictor of its persistence into adulthood [3]. A meta-analysis of 35 epidemiologic studies calculated that between 350,000 and 450,000 US children and adults have TS and estimated that the prevalence of TS together with other persistent tic disorders ranges from 560,000 to almost 1.7 million persons worldwide, with males being 3–4 times more likely to receive such a diagnosis [4,5]. As many as half of these individuals with TS are estimated to have not been identified or diagnosed by a healthcare professional [6].

TS is highly associated with co-morbid disorders, notably obsessive compulsive disorder and attention deficit hyperactivity disorder, and some individuals also have deficits in their cognitive development [7].

Tourette syndrome can have severe consequences, not only from the intrusive and disruptive nature of the tics but also from the social stigma others associate with anomalous behaviors. As with so many neuropsychological conditions, there is a significant need for new, safer, better-tolerated, and effective pharmacotherapies to treat patients with TS.

Dysfunction of the basal ganglia is implicated in the neurobiology of TS [8,9], which provides a framework for developing new therapeutic approaches to the disorder. The basal ganglia are a network of subcortical nuclei that process input from the cortex on behavioral opportunities to feedback advantageous actions while suppressing competing actions [10,11]. These activities segregate into two coordinated processing streams: the direct pathway, mediating action selection, and the indirect pathway, mediating action suppression [12,13,14]. The input neurons of the basal ganglia are the striatal medium spiny neurons (MSNs), which merge the cortical glutamatergic input with a dopaminergic input from the substantia nigra and ventral tegmentum, which carries information related to expected reward and motivation [15,16]. The MSNs of the direct pathway express dopamine D1 receptors, whereas indirect-pathway MSNs express D2 receptors. The basal ganglia organize behaviors as ‘chunks’ that are adaptively expressed in action sequences [17,18]. A tic is the execution of a chunk outside of an adaptive sequence and may arise when a chunk gains an aberrant reward value [19,20]. This hypothesis is consistent with the interpretation that, in many individuals, tics are preceded by a premonitory urge and, upon execution, the tic returns a ‘reward’ that relieves the urge. However, it has also been suggested that tics result from an aberrant sensitivity to the triggering of a chunk [21,22]. These two possibilities are likely mutually reinforcing rather than exclusive.

Consistent with the above conceptualization of tics, a mainstay of FDA-approved pharmacological interventions in Tourette syndrome is dopamine D2 receptor antagonists [23]. By inhibiting D2 receptors, these agents activate the MSNs of the indirect striatal pathway to suppress the expression of tics. While these agents show substantial efficacy in clinical trials and clinical practice, they carry significant safety and tolerability liabilities. D2 antagonists can produce their own set of motor abnormalities, have significant metabolic adverse effects, and can cause neurocognitive and emotional disruption [24]. Thus, these agents must be used with great caution and restraint, particularly in children.

The inhibition of phosphodiesterase 10A (PDE10A) is a novel approach to the treatment of basal ganglia disorders [25,26,27], including TS. PDE10A is a member of the phosphodiesterase superfamily of enzymes that regulate through the metabolic inactivation of the intracellular second messengers cAMP and cGMP [28]. PDE10A is unique in that it is expressed at high levels only in MSNs and to a very limited extent elsewhere in the brain and body [29,30,31]. In MSNs, PDE10A regulates both cAMP and cGMP signaling [32], although the cyclic nucleotide pools under the control of the enzyme are not directly or exclusively linked to dopamine signaling [27]. Despite its expression in all MSNs, PDE10A inhibition results in the preferential activation of the indirect striatal output pathway [33,34,35], which, in some preclinical assessments, is evident as behavioral suppression similar to that caused by D2 antagonists [32,36].

Nonetheless, PDE10A inhibitors have a unique profile, possibly in part due to their coincident activation of the direct pathway and the indirect pathway [37,38,39]. This was captured in a study of the PDE10A inhibitor MP-10 in non-human primates, where D2 antagonism disrupted the motor expression of behavior, but PDE10A inhibition appeared to have suppressed the initiation of task execution [27,40].

PDE10A inhibitors are of interest as potential therapeutics for several basal ganglia function disorders but have also been tested in disorders with more diffuse pathophysiologies, such as schizophrenia and Huntington’s disease. PDE10A inhibitors have been most extensively investigated for their efficacy in patients with schizophrenia. Efficacy was not observed in phase 2 studies by Pfizer, Takeda and Lundbeck [41,42,43], however a recent phase 2 trial from Celon pharma reported efficacy in acute schizophrenia for CPL500-036, and another clinical trial is ongoing with Merck’s compound MK-8189 [44] due to finish in June 2024.

In a trial in Huntington’s disease, the Pfizer PDE10A inhibitor MP-10 did not show efficacy on the primary endpoints but was found to produce consistent and dose-dependent improvements in a quantitative motor (Q-motor) assessment [45]. There are also ongoing clinical studies of Noema Pharma’s PDE10A inhibitor NOE-105 for the treatment of TS and childhood-onset fluency disorder (stuttering).

Herein, we report the pharmacological characterization and nonclinical and clinical results of phase 1 human studies of EM-221 (previously known as MR-1916), a novel, highly potent, and selective PDE10A inhibitor under investigation for the treatment of TS, with additional potential indications under consideration.

## 2. Materials and Methods

EM-221 (MR1916) was provided by Mochida Pharmaceutical Co., Ltd. (Tokyo, Japan). For preclinical pharmacology studies, the compound was suspended in 0.5% methylcellulose solution (FujiFilm Wako Pure Chemical Industries, Ltd. Richmond, VA, USA) and administered orally at a volume of 5 mL/kg.

### 2.1. PDE10A Inhibitory Activity

The inhibitory potency of EM-221 against PDE10A was assessed using a FRET-based chemiluminescence assay. Recombinant full-length human PDE10A1 and PDE10A2, full-length mouse PDE10A2, and rat PDE10A catalytic domains were purchased from BPS Bioscience. Recombinant human, monkey, and dog PDE10A catalytic domains with N-terminal hexa- histidine tags were expressed in Escherichia coli strain BL21 (DE3) carrying the expression plasmid. The expressed PDE10A was purified on columns of HisTrap HP and Superdex75 10/300 GL (GE Healthcare). The inhibition of PDE10A by EM-221, MP-10, and TAK-063 was determined using the IMAP TR-FRET Screening Express system (Molecular Devices). The assay was performed in a 384-well white plate (Corning) at room temperature. Each recombinant PDE10A was preincubated with a test compound for 5 min prior to the addition of substrate, fluorescein-labeled cAMP, followed by incubation for 1 h (except for human PDE10A2, which was incubated for 40 min). IMAP binding solution was added and incubation continued for a further 3 h. TR- FRET chemiluminescence from each well was measured using an ARVO-Sx plate reader (Perkin Elmer). The maximal inhibition (100% activity) was defined as the no-enzyme control and no inhibition (0% activity) was defined as the no-compound control. Each assay was performed in quadruplicate at 8-test compound concentrations.

### 2.2. PDE10A Selectivity

The IC50s of EM-221 for the inhibition of representatives from each of the 11 PDE families were obtained using the Eurofin Panlabs PDE Selectivity Screen, which includes PDE 1A, 2A, 3A, 4A, 4B, 5A, 6, 7A, 7B, 8A 9A, 10A, and 11A.

The off-target effects of EM-221 were assessed using the Cerep BioPrint^®^ (Levescault, France) in vitro pharmacology battery, which consists of 104 radioligand binding assays (including non-peptide, peptide, and nuclear receptors; ion channels; and amine transporters) and 32 enzymes assays (including kinases, proteases, and phosphodiesterases). The concentration of EM-221 screened was 10 μM. At this concentration, only A1 adenosine receptors exhibited an inhibition of ligand binding greater than 50%. As a follow-up, functional agonist and antagonist activities against adenosine A1, A2A, and A3 receptors were determined.

### 2.3. In Vivo Activities in Rat

All animal studies were conducted in compliance with local laws in Japan and the US governing the ethical use and treatment of experimental animals for each of the labs that conducted testing.

### 2.4. Determination of EM-221 in Plasma and Striatum

EM-221 was administered via the oral route and at different times; thereafter, rats were sacrificed by decapitation and their blood and brains were collected. Plasma was isolated from the blood samples by centrifugation. Following brain isolation, the striatum was dissected bilaterally, weighed, and homogenized in a volume of 10 mL of ice-cold buffer (25 mM Sodium Phosphate, pH 7.4) using a Teflon homogenizer. All samples were frozen at −80 °C until their EM-221 levels were determined by LC-MS/MS.

### 2.5. PDE10A Enzyme Occupancy

To assess brain exposure and the PDE10A target engagement of EM-221, an in vivo enzyme occupancy assay was developed in rats using [3H]-PDM-042 as a PDE10A-selective radiotracer.

PDM-042 is a prototype PDE10A-selective inhibitor with good CNS penetration which is suitable as an in vivo radioligand [46]. [3H]-PDM-042 (662 GBq/mmoL) was synthesized by Sekisui Medical Co., Ltd. (Sapporo, Japan)

EM-221 at a dose of 0.1 mg/kg p.o. was administered orally and animals were sacrificed at 1, 2, 4, 8, 12, 16, and 24 h afterwards by decapitation (n = 3 rats per time point). Ten min prior to sacrifice, [3H]-PDM-042 was administered intravenously (3 µCi/rat). Following brain isolation, the striatum was dissected bilaterally, weighed, and homogenized in a volume of 10 mL of ice-cold buffer (25 mM Sodium Phosphate, pH 7.4) using a Teflon homogenizer. Homogenates (5 mL) were then filtered through 0.3% polyethylenimine-soaked Whatman GF/B filters and washed twice with a volume of 5 mL of ice-cold homogenization buffer. The filters were immersed in 3 mL of scintillation fluid, and radioactivity was counted on a Tri-Carb 291OTR scintillation counter (Perkin Elmer, Waltham, MA, USA). Non-specific binding was defined as the radioactivity measured in striatal tissue taken from rats administrated PDM-048 at a dose of 30 mg/kg p.o.

### 2.6. Effects of cAMP and cGMP in the Striatum

Rats were administered EM-221 via the oral route and 2 h later animals were sacrificed by focused microwave irradiation of the head (MMW-05, Muromachi Kikai Co., Ltd., Tokyo, Japan). The striatum was dissected, weighed, and then frozen in liquid nitrogen. For cyclic nucleotide measurements, frozen striatal tissue was homogenized in 5% trichloroacetic acid and centrifuged and the supernatant was collected. Supernatants were ether-washed and then the cAMP and cGMP levels in the aqueous phase were determined by enzyme immunoassay using kits from Cayman Chemical Co. (Ann Arbor, MI, USA)

### 2.7. Effects on Enkephalin and Substance P mRNA Expression in the Striatum

Rats, habituated to the test room for at least 1 h, were administered EM-221 (0.025–0.8 mg/kg p.o. or vehicle) and then sacrificed after 2, 3, or 4 h by decapitation (n = 4/group). Following brain isolation, the striatum was dissected bilaterally and immediately frozen in liquid nitrogen. Samples were stored at −80 °C until use. Total RNA from striatum was isolated using QIAzol reagent and an RNeasy Mini Kit according to the manufacturer’s instructions (QIAGEN, Hilden, Germany). The concentration of the total RNA was determined by NanoDrop spectrophotometry (Thermo Fisher, Waltham, MA, USA). For a quantitative real-time PCR analysis, 2 µg of total RNA was converted to cDNA in a 20 µL reaction buffer using a Superscript VILO cDNA Synthesis Kit according to the manufacturer’s instructions (Thermo Fisher-Life Technologies, Waltham, MA, USA). The cDNA was diluted 50-fold with distilled water, and 2 µL was used per PCR reaction. TaqMan Gene Expression Assays (FAM) from Life technologies were used to quantify the EM-221-induced change in expression of enkephalin (assay ID Rn00567566) and substance P (assay ID Rn01500392).

The expression of hypoxanthine phosphoribosyltransferase 1 (assay ID Rn0l500392) served as the control to correct for differences in the assays’ total mRNA.

### 2.8. Inhibition of MK-801-Induced Hyperlocomotion

The locomotor activity in rats in an open field (plastic cage of 270 mm width × 440 mm length × 187 mm height lined with clean paper chips) was quantified using a Supermex activity monitoring system (Muromachi Kikai Co., Ltd., Tokyo, Japan). Their locomotor activity was measured for 1 h immediately after EM-221 administration. Rats were habituated to the test room for at least 1 h before testing. EM-221 was administrated at doses of 0.01–0.10 mg/kg p.o. (n = 8/group), after which the animals were immediately placed into the open field and their locomotor activity was measured for 1 h (spontaneous locomotor activity). Animals were briefly removed from the open field for the administration of MK-801 (0.2 mg/kg, s.c.) and then their locomotor activity was measured for a further 2 h period (MK-801-induced hyperlocomotion).

### 2.9. Inhibition of Conditioned Avoidance Responding

The conditioned avoidance response test [47] was conducted in shuttle boxes enclosed in sound-attenuating chambers (MED Associates, Fairfax, VT, USA). The conditioned avoidance response test is a sensitive test for the detection of potential atypical antipsychotics. The Plexiglas shuttle boxes were divided by a guillotine door into two compartments. The floor of each compartment comprised a series of metal grid rods for the delivery of scrambled electric foot shocks. Each side of the shuttle box was equipped with a stimulus light, speaker to deliver a tone, and infrared beam detectors to locate the animal within the box.

Rats were trained to associate the stimulus light and tone with the delivery of a foot shock. Animals learned to avoid the foot shock by shuttling from the compartment with the light and tone to the other compartment. Training consisted of daily sessions, each of 30 trials. Each trial consisted of a 10 s presentation of the stimulus light and tone in the compartment in which the animal was located (conditioned stimulus; CS) followed by a foot shock of 0.8 mA for 10 s (unconditioned stimulus; UCS). Crossing to the opposite compartment constituted an escape, which triggered an inter-trial interval of a randomized duration, between 7.5 and 22.5 s, before the next trial. During daily training sessions, animals learned to avoid foot shocks by traversing to the opposite compartment after the onset of the CS but prior to the UCS. Animals were required to achieve more than 80% avoidance of the UCS for 3 consecutive days prior to drug testing.

Animals that met the training criteria were assigned to dose groups balanced for avoidance rates. EM-221 was administered at doses of 0.025 to 0.2 mg/kg p.o. 2 h before test sessions. A test session consisted of 30 CS-UCS trials separated by inter-trial intervals. The number of trials in which the animal avoided shock, escaped shock, or failed to respond were recorded.

### 2.10. Novel Object Recognition

The novel object recognition test was conducted in an open-field box (length, 60 cm; width, 60 cm; height, 35 cm) made of gray-colored polyvinylchloride with a floor covered with sawdust in a dimly illuminated room. The objects to be discriminated were transparent glass bottles (with a blue cap, 15 cm height) and brown glass bottles (with a brown cap, 15 cm height). The two objects were placed in a symmetrical position about 10 cm away from the wall.

One day before the acquisition trial, rats were allowed to explore the field for 10 min (habituation) without objects. During the acquisition trial, a rat was placed in the experimental apparatus, facing the wall at the opposite end from the objects, and then the rat was allowed to explore the two identical objects. Exploratory behavior was monitored for 3 min using a video camera mounted above the experimental apparatus. Recordings were scored offline by a trained observer who was unaware of the treatment conditions. The exploration of an object was defined as the rat pointing the nose to an object at a distance of <1 cm and/or touching it with the nose. Turning around or sitting on an object was not considered exploration. The rat was then removed from the apparatus and test agents were administered, after which the animals were returned to their home cage. The post-acquisition administration paradigm was chosen to avoid the effect of drugs on animal behavior during the acquisition period. Sawdust was stirred and the objects were thoroughly cleaned with 70% ethanol after each trial.

The test trial was performed 48 h after the acquisition trial. In the test trial, one copy of the familiar objects explored during the acquisition trial was replaced by a new object. All combinations and locations of objects were balanced to reduce potential bias due to preferences for particular locations or objects. Exploratory behavior was again captured for 3 min by a video camera and scored offline.

A recognition index (RI) for the test trial was calculated as the ratio of the time spent exploring the novel object to the total time spent exploring the two objects. Data were excluded for (1) rats spending less than 10 s in total exploration during either the acquisition or test trial or (2) rats spending less than 1 s exploring one of the objects in either the acquisition or test trial.

### 2.11. Reversal of MK-801 Disruption of Prepulse Inhibition of Startle (PPI)

An SR-LAB startle chamber (San Diego Instruments, San Diego, CA, USA) was used for recording PPI. The SR-LAB software (SR-9020) controlled and delivered all the acoustic stimuli and recorded startle responses. Startle chambers featured a continuous 65 dB background white noise and a continuously running fan. To obtain stable startle responses, animals were acclimated to the test chamber for 5 min and then preconditioned by the delivery of three startle stimuli (40 msec bursts of white noise at 120 dB). A test session consisted of 3 blocks of 5 repetitions of startle sequences. PPI was induced by 20 msec sound pulses of 70, 75, or 80 dB delivered in the 100 msec prior to the 120 dB startle pulse. Controls in separate blocks included responses to the 120 dB startle pulse alone, to the 20 ms 80 dB prepulse alone, and when no stimulus was presented. The trial blocks were presented in pseudo-random order, with inter-trial intervals averaging approximately 15–30 s. Fifteen responses to the same stimulus sequence were averaged and used for the calculation of PPI as a percentage of the startle amplitude using the formula

$$\frac{\left(\mathrm{startle}\;\mathrm{amplitude}\;\mathrm{alone}-\mathrm{startle}\;\mathrm{amplitude}\;\mathrm{when}\;\mathrm{proceded}\;\mathrm{by}\;\mathrm{prepulses}\right)}{\mathrm{startle}\;\mathrm{amplitude}\;\mathrm{alone}}\times 100$$

PPI was disrupted by the subcutaneous administration of 0.1 mg/kg MK-801 15 min before test sessions. A number of drugs were administered prior to MK-801 to determine its efficacy to reverse the MK-801-induced disruption of PPI. These were the PDE10A inhibitor EM-221 (0.1, 0.3, and 1 mg/kg p.o.) or aripiprazole (10, 30, and 100 mg/kg p.o.) administered 2 h before testing; the PDE10A inhibitor MP-10 (3, 10, and 30 mg/kg p.o.) or TAK-063 (0.3, 1, and 3 mg/kg p.o.) or lurasidone (10, 30, and 100 mg/kg p.o.) administered 1 h before testing; or risperidone (0.1, 1, and 3 mg/kg p.o.), olanzapine (1, 3, and 100 mg/kg p.o.) or clozapine (1, 3, and 100 mg/kg p.o.) administered 45 min before testing.

### 2.12. Clinical Studies

Single ascending dose (SAD), multiple ascending dose (MAD), and PET studies were conducted at a single site to evaluate safety, tolerability, pharmacokinetics, and enzyme occupancy in healthy adult subjects under fasted or fed conditions. Subjects were assigned to drug or placebo under double-blind randomization in successive cohorts of 6 on drug and 2 on placebo.

In the SAD study, 6 cohorts were enrolled at doses from 0.25 mg to 15 mg. Subjects were evaluated as inpatients for 3 days, as outpatients for 3 additional days, and then at follow-up visits at days 10 and 14 and during a telephone follow-up between days 21 and 28 after dosing.

In the MAD study, 3 cohorts were enrolled and dosed at 5 mg, 10 mg, or 15 mg. Subjects received drug once daily for 8 days under fed conditions (after a normal breakfast) and were evaluated as inpatients through to day 13 after the first dose. A follow-up visit was conducted between days 10 and 14 after discharge and a telephone follow-up at days 21–28 after initial dosing.

In both studies, evaluations consisted of multiple blood draws for analysis of plasma drug levels and routine laboratory assessments, including determination of plasma prolactin levels. General safety was monitored through ECG assessments, clinical safety assessments, and physical examinations.

Assessments more directly relevant to PDE10A inhibition included questionnaires to evaluate alertness, calmness, and contentedness (Bond and Lader visual analogue scales [48]); extrapyramidal symptoms (Extrapyramidal Symptom Rating Scale-abbreviated: ESRS-A [49]); sleepiness (Stanford sleepiness scale: SSS [50]); and suicidality (Columbia-Suicide Severity Rating Scale: C-SSRS [51]).

The calculated pharmacokinetic parameters for the SAD (all doses) and MAD studies (first and last 24 h) were time to maximum observed plasma drug concentration (t_max_), maximum observed plasma drug concentration (C_max_), area under the plasma concentration time curve from time 0 to the last sampling time (AUC_0–t_), and area under the plasma concentration time curve from time 0 to infinity (AUC_0–∞_).

Evaluation of the occupancy of PDE10A by EM-221 was estimated from PET imaging of [^11^C]–IMA107 [52]. Subjects were 6 healthy males who received a single oral dose of 2 mg, 10 mg, or 15 mg under fasted conditions followed by a bolus intravenous dose of the PET ligand. Subjects underwent 2 or 3 scans at 2, 7, 26, or 31 h post-dose. For the quantification of [^11^C]–IMA107 data, regional time–activity curves were extracted from the PET images by fitting to the simplified reference tissue model (SRTM). Values for the change in binding potential relative to the non-displaced component (ΔBP_ND_) were calculated for the target regions of interest (ROI): dorsal caudate (DCa), dorsal putamen (DPu), and accumbens (Acc). The cerebellum was used as the reference region.

ΔBP_ND_ data were plotted against measured plasma concentration data for EM-221, and the following model was fitted to determine PDE10A occupancy:

$$\Delta BP_{ND}=E_{max\;}\times \frac{C_{p}^{n}}{C_{p}^{n}+EC_{50}^{n}}$$

where *C_p_* is the measured plasma concentration of EM-221 (ng/mL), *E_max_* is the maximal apparent achievable change in BP_ND_ (%), *EC_50_* is the plasma concentration of EM-221 that corresponds to 50% of that maximal change in BPND (ng/mL), and n is the Hill slope. Variants of the model were considered whereby *E_max_* and *n* were either fitted or fixed (100% and 1, respectively). Model selection was performed via the consideration of the residual sum of squares and the Akaike Information Criterion (AIC). Where *E_max_* is 100%, the ΔBP_ND_ data can be considered as target occupancy; otherwise, the occupancy is given by *Occ* = *ΔBP_ND_/E_max_*.

## 3. Results

### 3.1. Preclinical Pharmacology

#### 3.1.1. In Vitro Assessments of PDE Potencies and Selectivity

EM-221 exhibits inhibitory activities towards recombinant full-length human PDE10A1 and PDE10A2 enzymes, with half-maximal inhibitory concentration (IC_50_) values of 8.9 and 12 pM, respectively. The IC_50_ values of EM-221 for recombinant human, rat, monkey, and dog PDE10A catalytic domains are 22, 20, 22, and 15 pM, respectively. EM-221, at 10 μM, does not exhibit inhibitory effects >50% against representatives from the other 10 PDE families, except for human PDE5A, at 58% inhibition.

Furthermore, EM-221 at 10 μM has minimal off-target pharmacological effects on the 136 molecular targets of the Cerep BioPrint^®^ in vitro screening battery. In this screen, the only other target besides PDE10A that was positive was the adenosine A1 receptor, with an IC_50_ value of 7.6 μM.

#### 3.1.2. Pharmacokinetics, Brain Penetrability, and Receptor Occupancy in Rodents

**Figure 1:**
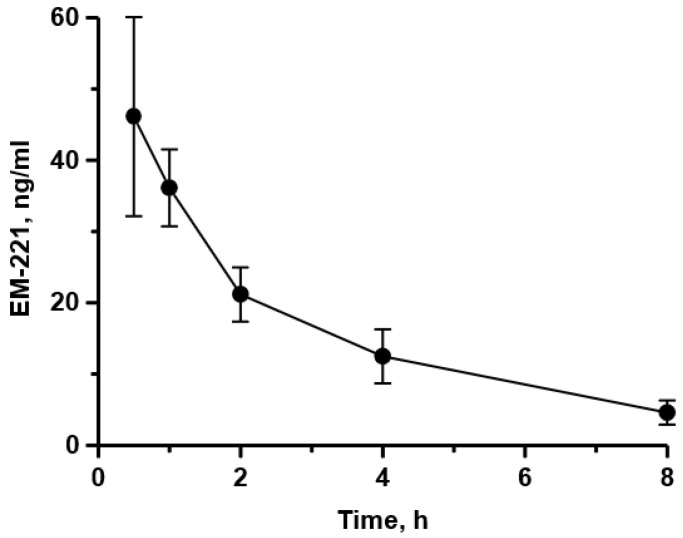
EM-221’s plasma concentration time course after its oral administration at 0.3 mg/kg in rats.

Figure 1 depicts the time course of plasma exposure in male rats following an oral dose of 0.3 mg/kg EM-221, a dose that was near the maximal efficacy in functional studies. EM-221 was well absorbed and a plasma C_max_ of 46 ng/mL was achieved at a tmax of 30 min. Plasma levels decreased by ~50% at 2 h and by ~75% at 4 h after oral dosing, indicating a half-life of approximately 2 h. Plasma exposure was linear over the range of efficacious doses in rat, based on exposures measured at 2 h after 0.025 mg/kg through 0.2 mg/kg oral doses (Figure 1). Interestingly, the levels of EM-221 in the striatum, expressed as ng/g tissue, were more than 20–50-fold higher than its level in plasma (Table 1).

**Figure 2:**
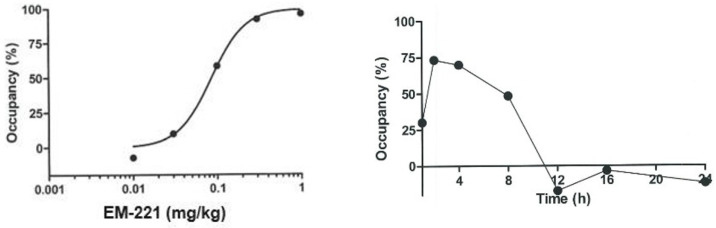
In vivo PDE10A occupancy in the rat striatum after oral administration. **Left** panel—PDE10A enzyme occupancy at 2 h after different doses of EM-221 p.o. **Right** panel—PDE10A occupancy as a function of time after an oral dose of 0.1 mg/kg EM-221. Each point is the mean of data collected from 3 rats.

The blood–brain barrier penetration of EM-221 was further assessed as the ability of systemic administration to displace the PDE10A radioligand [^3^H]-PDM-042 in measurements of striatal enzyme occupancy (see Section 2.5). EM-221 administration caused a dose-dependent reduction in [^3^H]-PDM-042 binding to PDE10A in the striatum, with a calculated 96.4% occupancy at a dose of 1 mg/kg p.o. (Figure 2, left panel). After an oral dose of 0.1 mg/kg, PDE10A occupancy by EM-221 reached its highest level at 2 h post-dose and decreased in a time-dependent manner between 2 h and 12 h post-administration (Figure 2, right panel). It is noted that the time course for the decrease in PDE10A occupancy was slower than the decrease in plasma levels (compare Figure 1 and Figure 2, right panel). This observation, as well as its high striatal levels relative to plasma levels, suggests that EM-221 may have a slow dissociation from the enzyme after binding, consistent with the very high affinity of the compound for PDE10A determined from in vitro studies.

#### 3.1.3. Pharmacodynamic Activities in Rats

**Figure 3:**
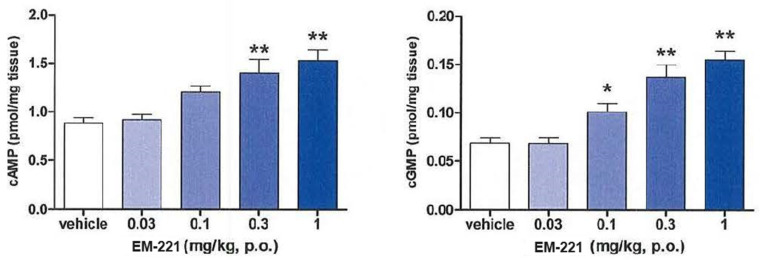
Levels of cAMP and cGMP in striatum (pmol/mg tissue) at 2 h after different oral doses of EM-221. Asterisks represent statistical differences from the vehicle group: * *p* < 0.05, ** *p* < 0.01.

At 2 h after oral doses of EM-221 between 0.03 and 1.0 mg/kg, there was an increase in striatal levels of cAMP and cGMP (Figure 3). The magnitude of the maximal increases induced by 1 mg/kg of EM-221 was similar to the increases induced by a maximal dose of the Pfizer PDE10A inhibitor MP-10 (30 mg/kg).

EM-221 significantly increased levels of messenger ribonucleic acid (mRNA) in the striatum for enkephalin at doses > 0.1 mg/kg p.o. and for substance P at doses > 0.2 mg/kg p.o. This is indicative of the fact that EM-221 activated the MSNs of the indirect and direct pathways, respectively.

EM-221 generated pharmacodynamic activities in rat behavioral assays that are consistent with PDE10A inhibition and in dose ranges consistent with the enzyme occupancy measurements. EM-221 reduced hyperlocomotion induced by MK-801 (0.2 mg/kg, s.c.), with an ED50 between 0.03 and 0.1 mg/kg p.o.

EM-221 also attenuated conditioned avoidance responses in rats over a similar dose range of 0.025–0.2 mg/kg p.o., with an ED50 value of 0.042 mg/kg p.o. In the novel object recognition test (ORT) in rats, EM-221 dose-dependently increased the recognition index at all doses > 0.1 mg/kg p.o. Pretreatment with EM-221 tended to attenuate the disruption of prepulse inhibition of startle (PPI) induced by MK-801 (0.1 mg/kg s.c.) at all prepulse intensities (70, 75, and 80 dB). The effects of EM-221 on the induction of catalepsy were variable and not clearly dose-dependent. Over a dose range of 0.05-0.8 mg/kg p.o., EM- 221 significantly induced catalepsy only at the dose of 0.2 mg/kg p.o. in rats.

#### 3.1.4. Preclinical Safety Assessment

In the dose range of 0.1–1 mg/kg p.o., EM-221 did not affect plasma prolactin concentrations nor increase plasma glucose levels following an intraperitoneal glucose tolerance test (ipGTT).

A standard battery of regulatory compliant safety pharmacology and genotoxicity studies were conducted in support of an Investigational New Drug Application. The results of all genotoxicity studies were unremarkable. In the safety pharmacology studies, EM-221 produced acute, moderate, and transient CNS depression (a decrease in locomotor activity, incomplete eyelid opening, low arousal, prone position, and/or deep respiration) in rats at doses of 0.03 mg/kg and above and minimal decreases in respiration rate at doses of 10 mg/kg. In dogs, EM-221 produced transient slight increases in heart rate at doses of 0.3 mg/kg and above.

A series of regulatory compliant toxicological studies, up to 91 days in duration, have been conducted with EM-221 to investigate its toxicological profile. Rats and dogs were chosen as the primary toxicology species based on the need to conduct repeat-dose studies in rodent and non-rodent species to support clinical trials. Human, rat, and dog hepatic microsomes produced similar cross-species metabolite profiles.

EM-221 was well tolerated at all doses administered to rats and dogs, and a clear adverse effect level was not established in any study. Following acute dosing in both rats and dogs, a brief period of inappetence and decreased weight gain was noted. In dogs, a slight tremor, salivation, tachypnea, and/or head weaving appeared approximately 0.5 to 6 h after the initial dose. Most subjective observations resolved by 8 h after the initial dose and did not recur during the study. Rats tolerated daily doses of 0.3 to 300 mg/kg (top evaluated dose) and dogs tolerated doses of 1 to 100 mg/kg (top evaluated dose) for up to 91 days without any significant alterations in clinical chemistries, hematology, or histopathology. Systemic exposures associated with the administration of 300 mg/kg/day in rats for 91 days included a C_max_ value of 13,500 ng/mL and an AUC_24_ of 167,000 ng*h/mL. Systemic exposures associated with the administration of 100 mg/kg/day in dogs for 91 days included a C_max_ value of 5050 ng/mL and an AUC_24_ of 54,000 ng*h/mL. These exposures represent substantial multiples of those expected to be required for a therapeutic effect.

### 3.2. Clinical Results

#### 3.2.1. SAD Study Pharmacokinetics

**Figure 4:**
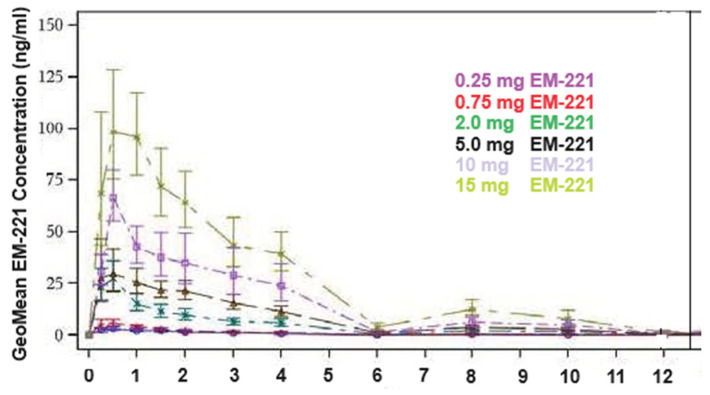
Single-dose plasma concentration time profiles (linear scale) by treatment dose in humans.

Following a single oral dose of 0.25–15 mg, plasma concentrations rapidly increased, suggesting absorption in the upper gastrointestinal tract, with a median t_max_ ranging between 0.38 and 0.49 h post-dose (Figure 4). Elimination was consistent with first-order kinetics. Plasma exposure was somewhat less than dose-proportional across the 0.25 to 15 mg dose range for C_max_ and AUC_0-∞_; however, the estimate for AUC_0-t_ was considered dose-proportional at 0.88 (Confidence interval = [0.732; 1.028]). The median apparent terminal half-life (t_1/2_) was between 9.06 and 13.5 h.

#### 3.2.2. MAD Study Pharmacokinetics

**Figure 5:**
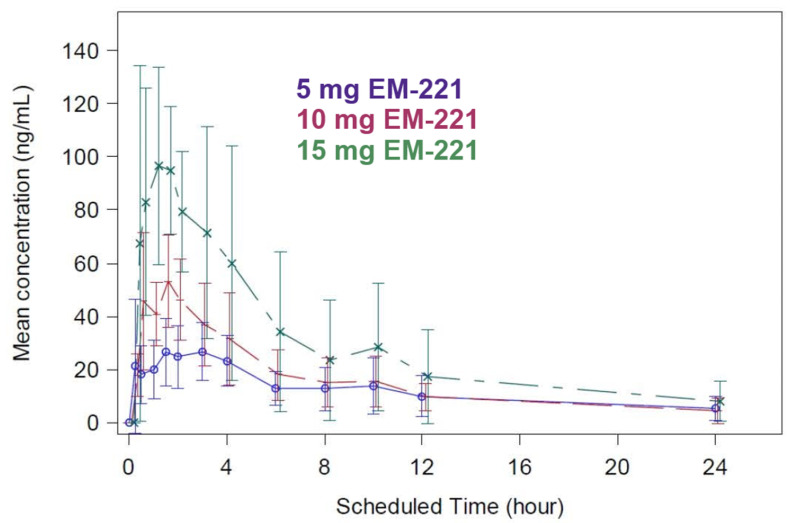
Multiple-dose plasma concentration time profiles (linear scale) by treatment dose in humans, day 1 (dosed with food).

Absorption was rapid, with a median t_max_ of 0.75–1.63 h. PK on day 1 and elimination was consistent with findings in the SAD study, with a median terminal t_1/2_ on day 8 of 10–15 h (Figure 5). C_max_ was approximately dose-proportional. The geometric mean AUC_24_ increased 2.3-fold over a 3-fold increase in dose, which was less than dose-proportional. However, this may be due to a very small sample size in the 15 mg group due to three subjects being withdrawn due to vomiting. There was little to no accumulation over the 8 days of dosing.

The geometric mean steady-state AUC_inf_ and AUC_last_ in the 10 mg dose group was lower than those parameters in the 5 mg dose group, but this finding was driven by outlier data from a single subject. When this outlier was removed, the data were consistent with the expected dose-proportional increase in exposures.

#### 3.2.3. PET Study Results

**Figure 6:**
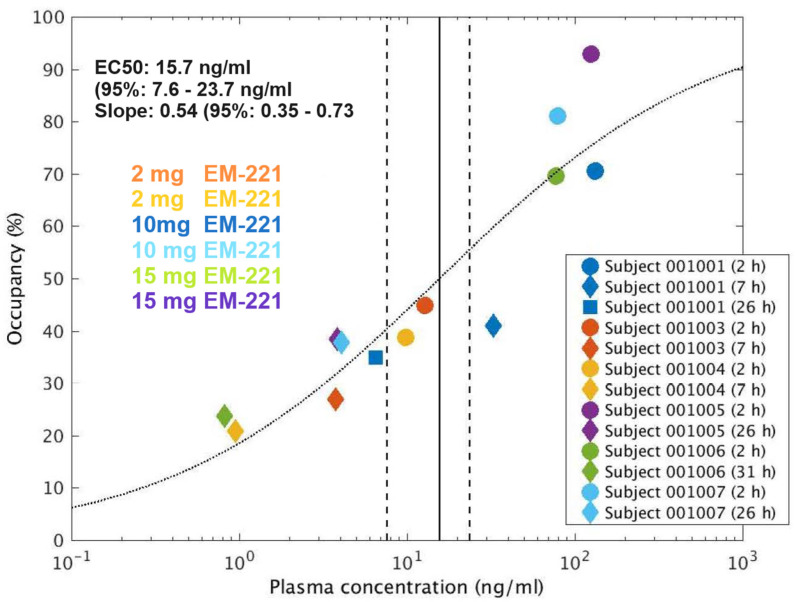
Global ΔBPND data plotted against measured plasma concentrations in humans.

PET images displayed the expected known distribution of PDE10A. Kinetic modeling produced acceptable model fits consistent with reduced target availability post-dose and well-determined binding potential (BP_ND_) parameters. Global ΔBP_ND_ values (the mean of the ΔBP_ND_ values for the target ROIs) are plotted against measured EM-221 plasma concentrations (Figure 6) and show a clear increase in ΔBP_ND_ with increasing concentration. The reduction in BP_ND_ over the measurement period was also calculated and was approximately 50% over a 24-hour period (Table 2). The model with fixed E_max_ (100%) and a fitted Hill slope was selected as the most appropriate model. This model produces an estimated EC50 of 15.7 ng/mL (95% confidence interval: 7.6–23.7 ng/mL), with a Hill slope (n) of 0.54 (95% confidence interval: 0.35–0.73). The global ΔBP_ND_ values fitted with this model can be considered PDE10A occupancy estimates.

#### 3.2.4. SAD Study Safety Results (Table 3)

EM-221 was safe and generally well tolerated across the dose range of 0.25–15 mg, with no serious or severe adverse events (AEs) and with AEs that were mostly mild. There were no clinically significant abnormalities in vital signs or laboratory or ECG findings. The most common AEs were somnolence (reported by 33.3% of subjects on the drug vs. 33.3% on placebo) and fatigue (19.4% vs. 0% on placebo) across the doses tested. The majority of AEs were reported at the maximum tolerated dose of 15 mg.

**Table 3:** Adverse events related to study treatment, by dose and study, in SAD and MAD (dosed once daily for 8 days).

	0.25 mg N = 6 n (%) E	0.75 mg N = 6 n (%) E	2 mg N = 6 n (%) E	5 mg N = 6 n (%) E	10 mg N = 6 n (%) E	15 mg N = 6 n (%) E	Placebo N = 12 n (%) E	Total (Subjects on EM-221)
**SAD** **study**	2 (33.3) 4	5 (83.3) 9	3 (50.0) 3	5 (83.3) 8	5 (83.3) 14	6 (100) 27	5 (41.7) 5	31 (83.3) 70
**MAD** **study**				4 (66.7) 12	3 (50%) 9	5 (83.3%) 35	5 (83.3%) 7	12 (94.4%) 56

N = number of subjects per cohort included in the Safety Set; n = number of subjects experiencing adverse events per cohort; E = number of events per adverse event.

#### 3.2.5. Neurological Adverse Events at the Highest Dose of 15 mg in the SAD

Several subjects in the supratherapeutic, high-dose 15 mg group showed mild–moderate neurological AEs on the extrapyramidal symptom rating scale-abbreviated (ESRS-A) exam, consistent with a high degree of basal ganglia D2 pathway inhibition, including mild to moderate dystonia, trismus, clenching of teeth, akathisia, bradykinesia/stiffness, and involuntary tongue protrusions, all of which resolved within 24 h. One subject given 10 mg had an intention tremor of the mouth and a mild tongue tremor around 6 h post- dose.

#### 3.2.6. MAD Study Safety Results (Table 3)

There were no severe or serious AEs, and all were mild to moderate in severity (one AE of moderate anxiety and two of moderate restlessness in the 5 mg group). There were no clinically significant findings in terms of vital signs, laboratory values, ECGs, the Bond Lader scales, the SSS (sleepiness), or the C-SSRS scale.

Regarding neurological AEs, musculoskeletal stiffness and muscle twitching were the most common neurological AEs. Stiffness was reported by two subjects in the 15 mg group and two subjects in the placebo group. Muscle twitching was reported by one subject in the 10 mg group and two subjects in the 15 mg group. Muscle tightness was noted in one subject in the 10 mg group and one subject in the 15 mg group, and trismus in one subject in the 10 mg group.

The doses of 5 mg and 10 mg were safe and well tolerated, and the percentage of subjects with drug-related TEAEs was higher in the placebo group (83.3%) than in the 5 mg (66.7%) and 10 mg (50.0%) groups.

The 15 mg dose was also safe but not well tolerated. Three subjects discontinued 15 mg dosing after a single episode of vomiting on days 2 or 3, with no clear predictive pattern in PK exposure: these subjects had the lowest, third lowest, and highest exposures within the group of six subjects on 15 mg. TEAEs were also higher in this group, with 21/36 AEs being reported by a single subject. The most common AEs across all doses were nausea, vomiting, and muscle twitching.

#### 3.2.7. PET Study Safety Results

Single oral doses (2, 10, or 15 mg) were well tolerated and had an acceptable safety profile. Notably, 15 mg was better tolerated than the 15 mg dose in the SAD and MAD studies. The most common TEAEs were somnolence and hot flush, and these both occurred at the lowest dose administered. There were no clinically relevant changes in any other safety parameters.

## 4. Discussion

### 4.1. Preclinical Pharmacology

In preclinical studies in rodents, EM-221 showed good brain penetration and activated the medium spiny neurons of both the direct and indirect striatal output pathways. In behavioral studies, EM-221 reduced MK-801-induced hyperlocomotion and conditioned avoidance responding, similar to antipsychotics. The compound also tended to restore deficits in prepulse inhibition of startle caused by MK-801 and facilitated novel object recognition. Efficacy was observed at 0.1 mg/kg p.o. in all behavioral assessments. This dose was estimated to result in a PDE10A occupancy of 60%. Finally, the occupancy of PDE10A persisted several hours beyond that expected from the plasma concentrations seen in rats, suggesting that EM-221 has a slow off-rate.

These results are consistent with EM-221 being a highly potent and specific PDE10A inhibitor. EM-221, like other PDE10A inhibitors, preferentially activates the indirect striatal output pathway [33,34,39]. In this respect, EM-221 has a pharmacological similarity to D2 antagonists, which are proven to have clinical efficacy in TS.

### 4.2. Nonclinical Toxicity

The evaluation of metabolite profiles of EM-221 generated from human, dog, and rat hepatic microsomal preparations were similar across species, indicating the relevance of rats and dogs to humans for safety assessments. Following oral dosing in both rat and dog toxicology studies, EM-221 achieved exposures more than 10-fold above those targeted for clinical use. Transient acute findings in rats and dogs were likely associated with exaggerated pharmacology rather than off-target toxicity. Preclinical lethargy, nausea, vomiting, and limited extrapyramidal symptoms (tremor, head weaving) resolved rapidly, often after the first dose, and did not recur with continued dosing. Clinical chemistries, hematology, and histopathology were unremarkable following repeat doses of up to 300 mg/kg in rats and 100 mg/kg in dogs for up to 91 days. These preclinical assessments indicate that the extended treatment of patients using EM-221 will likely be safe and well tolerated if initiated with titration to target dose.

### 4.3. Clinical

The pharmacokinetic, safety, and occupancy profiles of EM-221 support its continued development as a therapeutic. Exposures were proportional or somewhat less than proportional across a wide range of doses. Its absorption was rapid and there was no accumulation over 8 days of once-daily dosing. The calculated PK half-life was approximately 9–15 h across the two studies. In a PET study using [^11^C]–IMA107, an established PET ligand, to assess the PDE10A enzyme’s occupancy, peak occupancy occurred at 92.8% and correlated with peak plasma exposures, demonstrating that EM-221 effectively enters the brain and binds to the target in humans. Interestingly, and similar to the nonclinical occupancy finding, enzyme occupancy was decreased by only approximately 54% after 24 h and 66% after 31 h, which is significantly longer than that predicted if plasma exposures were highly correlated with enzyme occupancy. This uncoupling of exposure vs. occupancy may be reflective of a slow off-rate in the binding of EM-221 to the enzyme and suggests that the compound may be adequately dosed once daily.

The adverse effect profiles from the SAD and MAD studies were generally mild, similar to placebo, and relatively nonspecific in all but the highest dose of 15 mg given in a single dose with no titration or daily for 8 days, again with no titration. In the MAD study, there was only mild muscle twitching, tightness, and stiffness at 10 mg, and no neurological AEs at 5 mg. The 15 mg dose in the SAD study generated a number of extrapyramidal symptoms and findings, and the same dose in the MAD study, with no titration, caused vomiting in 3/6 subjects within 2–3 days. This high 15 mg dose also corresponds to a very high occupancy in the PDE10A enzyme of 70–98%.

These neurological extrapyramidal adverse effects are considered on-target pharmacology, confirming that the mechanism of action is activating the medium spiny neurons in the striatum. Given the similarities with the on-target motor effects of antipsychotics, this supports the hypothesis that EM-221, at a well-tolerated dose, may be beneficial for disorders in which neuroleptics are effective for hyperkinetic symptoms such as Tourette syndrome. Based on these data, doses at or below 10 mg will be safe and well tolerated, with a superior profile to that of antipsychotics, which is a critical point of differentiation. In the toxicology studies, adverse effects were transient even at high doses, supporting the clinical use of titration to minimize tolerability concerns. No clinically significant laboratory abnormalities were seen that would suggest metabolic dysregulation or increased cardiovascular risk, as is seen with antipsychotics, which is another important point of differentiation, since TS patients may require chronic dosing for consistent benefits.

A critical purpose of the phase 1 studies, beyond determining whether the drug is acceptably safe and well tolerated, was to inform dose selection and frequency of dosing for phase 2 studies in TS and other movement disorders. The totality of data from these phase 1 studies demonstrates that doses at or below 10 mg are safe and well tolerated and will engage with the PDE10A enzyme at levels that can reasonably be expected to improve hyperkinetic symptoms without generating neurological symptoms related to on-target pharmacology. Since occupancy of the target enzyme, with a T1/2 of ~24 h, rather than exposures per se are expected to predict clinical benefits, EM-221 can potentially be dosed once daily.

### 4.4. Tourette Syndrome

Currently, the FDA-approved treatments for TS are drugs that modulate dopamine signaling and that may be characterized as agents that increase the activation of the indirect striatal output pathway. These include the D2 antagonist haloperidol (approved in 1969 for adults and 1978 for children) and pimozide (approved in 1984) and, more recently, the D2 partial agonist aripiprazole (approved in 2014). Pimozide is rarely used due to its unacceptable safety risks. The clinical effectiveness of these drugs is shown at relatively high levels of D2 receptor occupancy: aripiprazole shows a D2 occupancy of ~85% at the indicated 10 mg daily dose [53], and haloperidol shows an occupancy of ~60–80% at the clinically effective doses of 2–5 mg daily [54]. Consequently, their efficacy comes with the burden of significant mechanism-based side effects. For the D2 antagonists, these include extrapyramidal symptoms such as dystonia, akathisia, and the potential for the development of tardive dyskinesia with long-term use. While the extrapyramidal symptoms are less of a burden with aripiprazole, both this drug and the D2 antagonists cause significant metabolic dysregulation, resulting in weight gain, insulin resistance, and overall long-term cardiovascular risk. For example, over 10 weeks, in a large, randomized trial, aripiprazole increased mean body weight, body mass index, and waist circumference significantly vs. placebo [55]. These safety liabilities are particularly concerning given that TS is a chronic pediatric disorder and that early metabolic disruption can have lifelong consequences.

The PDE10A inhibitor EM-221 offers a mechanistically novel approach to capturing the therapeutic efficacy of indirect pathway modulation. Because PDE10A inhibitors preferentially activate the indirect striatal output pathway [33,34,39], this class has pharmacological similarity to the currently used D2 modulators without engaging the D2 receptor. Nonetheless, there are differences between these two classes that may be of significance for this indication. It is noteworthy that, in primates, the PDE inhibitor MP-10 was found to dampen motivational aspects of behavior to a greater extent than a D2 antagonist [40]. Insofar as tics in TS are triggered by an aberrant reward drive to release a tic, this effect of PDE10A inhibition, if mechanism rather than drug specific, may contribute a unique component to efficacy in addition to the more direct suppression of tic release.

With regard to safety and tolerability, preclinical studies have identified important differences between PDE10A inhibitors and D2 antagonists. These differences have been found to be translational to humans in clinical studies with PDE10A inhibitors. PDE10A is not highly expressed in pituitary and EM-221 has not caused clinically meaningful increases in prolactin in preclinical rodent or human studies. More significantly, PDE10A genetic knock-out or pharmacological inhibition reduces the adverse metabolic consequences of high-fat diets in mouse models of metabolic syndrome and obesity [56]. The relevance of these findings to humans is indicated by the fact that the high-fat diets used to induce metabolic dysregulation are similar to a diet common to many in the US. Consistent with these preclinical findings, PDE10A inhibitors did not cause weight gain or induce other adverse metabolic sequalae in several phase 2 studies with multiple compounds, indicating a class effect. These data predict that EM-221 will have significant safety and tolerability advantages over the standard-of-care D2 modulators in TS patients, which may be particularly significant in children.

Other potentially treatable conditions with PDE10A inhibitors include schizophrenia, Huntington’s disease, L-dopa-induced dyskinesia in Parkinson’s disease, and childhood-onset fluency disorder.

PDE10A inhibitors have been and continue to be of interest in the treatment of other disorders in which basal ganglia dysfunction is implicated. The major interest has been in their use as potential antipsychotic agents in schizophrenia. However, in 4–6-week phase 2 studies of compounds from Pfizer, Takeda, and Lundbeck, there was no evidence of antipsychotic efficacy during acute exacerbations of schizophrenia [41,42,43,57]. There was also no additional benefit of Pfizer’s MP-10 as a adjunctive treatment with risperidone. The doses used in these trials would produce estimated occupancies of 20–50% where the data are available [58,59]. 

In contrast, Celon pharma recently reported positive efficacy vs. placebo and good safety and tolerability for their compound CPL500-036 for acute schizophrenia in a press release (Reference [44]). 

While the results to date of PDE10A inhibitors in schizophrenia have been mixed, these studies provide knowledge that may advance our understanding of the neurobiological basis of psychosis [27,60]. Effective antipsychotics show a wide range of target engagement, including dopaminergic, glutamatergic, serotonergic, muscarinic, and histaminergic circuitry [61]. Dopamine receptors are widely expressed in the CNS as D1, D5, and D2-4 families, both pre- and post-synaptically, playing complex roles in the mesocortical, mesolimbic, nigrostriatal, and tuberoinfundibular pathways [62]. Haloperidol, which binds to D2-4 but not D1, has activity across all dopaminergic pathways, and a precise understanding of their relative contributions to its efficacy for schizophrenia is still lacking. In contrast, PDE10A is highly localized to the striatum, which likely contributes to the differences in the safety and tolerability and efficacy of PDE10A inhibitors compared dopamine receptor antagonists. Their lack of efficacy in several studies to date in schizophrenia implies that broad modulation across multiple neurocircuitry pathways may be necessary for therapeutic efficacy in this poorly understood disease. The recent report of efficacy from Celon Pharma may point to differences across various PDE10A inhibitors, differences in dosing and engagement of the enzyme, or differences in the quality of the trial’s execution. There is an ongoing study of the PDE10A inhibitor MK-8189 in 500 patients with acute schizophrenia, estimated to finish in July 2024 (NCT04624243). If efficacious, the result together with the Celon Pharma results may re-kindle interest in the PDE10A mechanism as a novel and safer treatment approach than antipsychotics in this high-unmet-need population.

PDE10A inhibitors have also been studied for the treatment of Huntington’s disease. The degeneration of striatal MSNs is a cardinal pathology in Huntington’s disease and there are several lines of preclinical evidence suggesting that PDE10A inhibition may ameliorate this degenerative process [63,64].

However, in an initial 26-week phase 2 trial of Pfizer’s MP-10 vs. placebo in 272 patients with stage I–II HD and a Unified Huntington’s Disease Rating Scale-Total Motor Score (UHDRS-TMS, Huntington Study Group 1996) >10, there was no improvement in the primary outcome measure or the clinical global impression of improvement scale [45]. In contrast, a dose-dependent improvement in a pre-specified quantitative motor assessment (Q-motor) was found, which correlated with the UHDRS-TMS in other studies [65]. It is noteworthy that early in Huntington disease progression there is a decline in PDE10A expression levels in the striatum, as determined in post-mortem studies as well as with PDE10A PET imaging [66,67]. The decline in PDE10A expression putatively reflects medium spiny neuron pathology. It is unclear whether and how this loss of PDE10A impacts the potential efficacy of an inhibitor along the continuum of disease progression. At present, we are unaware of ongoing trials of PDE10A inhibitors for Huntington’s disease.

There are several other potential indications for PDE10A inhibitors. L-dopa-induced dyskinesias (LIDs) in Parkinson’s disease (PD) are a common, severe complication of L-dopa treatments in patients with more advanced PD. Studies in rodent models of LID have shown an association between lower cAMP/cGMP levels during the increasing phase of dyskinesias, which are prevented by amantadine treatment [68,69]. Amantadine, an NMDA antagonist, is the only approved therapy for LID and has both inconsistent effects and significant adverse effects including agitation, nausea, dizziness, insomnia, and a range of additional CNS and gastrointestinal adverse effects. 

EM-221 (MR1916) has been studied in the well-validated primate model of severe LID [70]. Five MPTP-treated macaques with advanced PD were treated with a range of doses (0.0015–0.05 mg/kg) in combination with L-Dopa, with amantadine as a positive control. Outcome was measured with a standard primate motor scale (PMS) [71] and the Drug Effects on the Nervous system (DENS) scale [72] to capture other changes in cortical, motor, and autonomic function. EM-221 significantly reduced LIDs with acute dosing similar to amantadine, with 0.015 mg/kg as the optimal dose, and with no reduction in the antiparkinsonian benefit of L-Dopa with chronic administration. There were no adverse effects observed at efficacious doses. Given the lack of treatment options for LID, an effective PDE10A inhibitor could meaningfully address the high unmet needs in this population. Celon Pharma has registered a phase 2 clinical trial to examine the efficacy of a PDE10A inhibitor in reducing L-dopa-induced dyskinesia, which is estimated to completein October 2024 (NCT05297201).

Childhood-onset fluency disorder (COFD) is a childhood-onset disturbance in the normal fluency of speech that persists over time and is inappropriate for the child’s age. Motor movements may accompany stuttering (tics, tremors, blinking, head jerking), and severity may be exacerbated by anxious anticipation. The underlying pathophysiology is complex and associated with aberrant network connectivity including the presence of dysfunctional circuits related to speech planning and the timing of initiation and execution of motor sequences [73,74]. Evidence also suggests that dopaminergic excess in the striatum may disrupt the cortico-basal ganglia-thalamocortical loop, which is further supported by the observation that L-dopa treatment in Parkinson’s disease may worsen stuttering [75]. There are no approved FDA treatments for COFD, but clinical trials and experience suggest that, like TS, antipsychotics are effective [76,77]. Other similarities to TS include childhood onset, high rates of comorbidity [78], predominance in males, a waxing and waning course, association with tic-like motor movements, and the growing body of evidence that COFD is related to dysregulation in the basal ganglia [79]. Evidence implicating basal ganglia dysregulation, and the fact that antipsychotics are effective for COFD, together with its similarities to TS, suggest the potential efficacy of a PDE10A inhibitor for COFD. There is an ongoing phase 2 trial of the PDE10A inhibitor NOE-105, sponsored by Noema Pharma, evaluating its efficacy in COFD (NCT05583955).

## 5. Conclusions

PDE10A inhibitors are a novel class of drug that continues to be explored for a wide range of indications (schizophrenia, Tourette syndrome, childhood-onset fluency disorder, L-dopa-induced dyskinesia in Parkinson’s disease) and may have potential applicability in others. The unusually specific neuroanatomical localization of the PDE10A enzyme to the basal ganglia suggests that its modulation is particularly relevant to diseases of striatal dysregulation. Continued research into modulating basal ganglia circuitry with this approach is critical to potentially meeting the high unmet need in all of these neurological conditions.

## Figures and Tables

**Table 1:** Plasma and striatum concentrations of EM-221 2 h after oral administration in rats.

Dose (mg/kg, p.o.)	Plasma (ng/mL)	Striatum (ng/g Tissue)	Striatum/Plasma
0.025	2.3 ± 0.3	124 ± 16	55
0.05	5.5 ± 1.2	237 ± 403	43
0.1	11 ± 2	348 ± 38	33
0.2	19 ± 1	416 ± 43	22

Data are mean ± SD, n = 3/dose.

**Table 2:** Binding-potential data by scan time post-dose and plasma concentration in target regions of interest.

Subject	Dose (mg)	Scan Time (Hours Post-Dose)	Plasma Conc. (ng/mL)	ΔBP_ND_ (%)				
				DCa	DPu	Acc	Mean	Reduction from 2 h to 26 or 31 h
1001	10	2	132	84.8	64.0	62.8	70.5	
		7	32.6	58.5	41.9	22.4	40.9	
		26	6.51	45.6	26.9	32.3	34.9	50.5%
1003	2	2	12.8	49.0	42.0	43.2	44.7	
		7	3.74	30.1	24.9	25.8	27.0	
1004	2	2	9.88	37.2	30.8	48.4	38.8	
		7	0.951	21.1	12.8	28.8	20.9	
1005	15	2	125	106.8	80.1	91.6	92.8	
		26	3.86	36.4	32.0	46.9	38.5	58.5%
1006	15	2	77.5	75.3	66.2	67.4	69.6	
		31	0.819	31.2	21.9	18.2	23.8	65.8%
1007	10	2	78.9	88.0	76.5	78.5	81.0	
		26	4.08	42.3	29.2	41.9	37.8	53.3%

Legend: dorsal caudate (Dca), dorsal putamen (DPu), and accumbens (Acc).

## Data Availability

The datasets presented in this article are not readily available because they are extracted from numerous large and proprietary study reports. Requests to access the datasets should be directed to the corresponding author.
